# Therapeutic Potential of Regulatory T Cells in Preeclampsia—Opportunities and Challenges

**DOI:** 10.3389/fimmu.2019.00478

**Published:** 2019-03-21

**Authors:** Sarah A. Robertson, Ella S. Green, Alison S. Care, Lachlan M. Moldenhauer, Jelmer R. Prins, M. Louise Hull, Simon C. Barry, Gustaaf Dekker

**Affiliations:** ^1^Robinson Research Institute and Adelaide Medical School, University of Adelaide, Adelaide, SA, Australia; ^2^University Medical Center Groningen, Groningen, Netherlands; ^3^Women's and Children's Hospital, Adelaide, SA, Australia

**Keywords:** pregnancy, preeclampsia, placenta, embryo implantation, maternal vascular adaptation, inflammation, Treg cells, immune tolerance

## Abstract

Inflammation is a central feature and is implicated as a causal factor in preeclampsia and other hypertensive disorders of pregnancy. Inflammatory mediators and leukocytes, which are elevated in peripheral blood and gestational tissues, contribute to the uterine vascular anomalies and compromised placental function that characterize particularly the severe, early onset form of disease. Regulatory T (Treg) cells are central mediators of pregnancy tolerance and direct other immune cells to counteract inflammation and promote robust placentation. Treg cells are commonly perturbed in preeclampsia, and there is evidence Treg cell insufficiency predates onset of symptoms. A causal role is implied by mouse studies showing sufficient numbers of functionally competent Treg cells must be present in the uterus from conception, to support maternal vascular adaptation and prevent later placental inflammatory pathology. Treg cells may therefore provide a tractable target for both preventative strategies and treatment interventions in preeclampsia. Steps to boost Treg cell activity require investigation and could be incorporated into pregnancy planning and preconception care. Pharmacological interventions developed to target Treg cells in autoimmune conditions warrant consideration for evaluation, utilizing rigorous clinical trial methodology, and ensuring safety is paramount. Emerging cell therapy tools involving *in vitro* Treg cell generation and/or expansion may in time become relevant. The success of preventative and therapeutic approaches will depend on resolving several challenges including developing informative diagnostic tests for Treg cell activity applicable before conception or during early pregnancy, selection of relevant patient subgroups, and identification of appropriate windows of gestation for intervention.

## Introduction

Preeclampsia and related hypertensive disorders complicate 3–5% of pregnancies. They are a leading cause of maternal deaths and perinatal morbidity and mortality (1) and are enormously expensive to health care systems, with an estimated cost in the US of $2.18 billion for the first 12 months of life alone (2). Preterm birth and fetal intrauterine growth restriction (IUGR) are common sequalae, causing developmental challenges for the neonate that adversely impact cardiovascular, metabolic, and neurodevelopmental health (3). Preeclampsia also has long-term consequences for maternal cardiovascular health (4). Despite extensive research, the pathophysiological origins of preeclampsia remain unclear and effective preventative interventions are lacking. Current clinical management is aimed at alleviating symptoms and delaying delivery, rather than preventing occurrence by modifying the underlying cause (5, 6).

An emerging view is that critical initiating events before pregnancy and in the conception and implantation phase determine preeclampsia susceptibility, eliciting changes in placental development much earlier in gestation than when symptoms appear (7–9). This is particularly the case for the severe, early onset form of preeclampsia where failed maternal vascular adaptation to pregnancy is implicated—but also likely contributes to later onset disease (10, 11). There is strong evidence that failure of the maternal immune response to adapt correctly in early pregnancy underpins the placental and cardiovascular anomalies that become evident in later gestation. Disturbance in the immune response appears to be central and causal of later placental and hypertensive symptoms (8, 12, 13).

The adaptive immune response, with its typical features of immunological priming and memory, appears integral to the pathophysiological origin of the condition. Preeclampsia is more common in first pregnancies, particularly after limited sexual contact with the conceiving partner due to short sexual cohabitation, use of barrier contraceptive methods or assisted reproduction (14–16). Prior pregnancy with the same partner offers protection, but this is partner-specific and is lost with a new partner, implying alloantigen specificity (17). Assisted reproduction with donor oocytes, where there is no prior contact with the donor's alloantigens, is associated with a 4.3-fold increase in preeclampsia compared to natural conception (18). The risk is also increased with donor sperm but this is reduced with multiple exposures to the same donor (19). Pregnancy-induced memory in T cells (20) and in uterine NK cells (21) likely contributes to the protective benefit of prior pregnancy, and mechanisms by which seminal fluid may also induce memory are emerging (22). Recognizing this protective role for the adaptive immune response offers scope for new approaches to tackle this prevalent condition.

**Figure 1:**
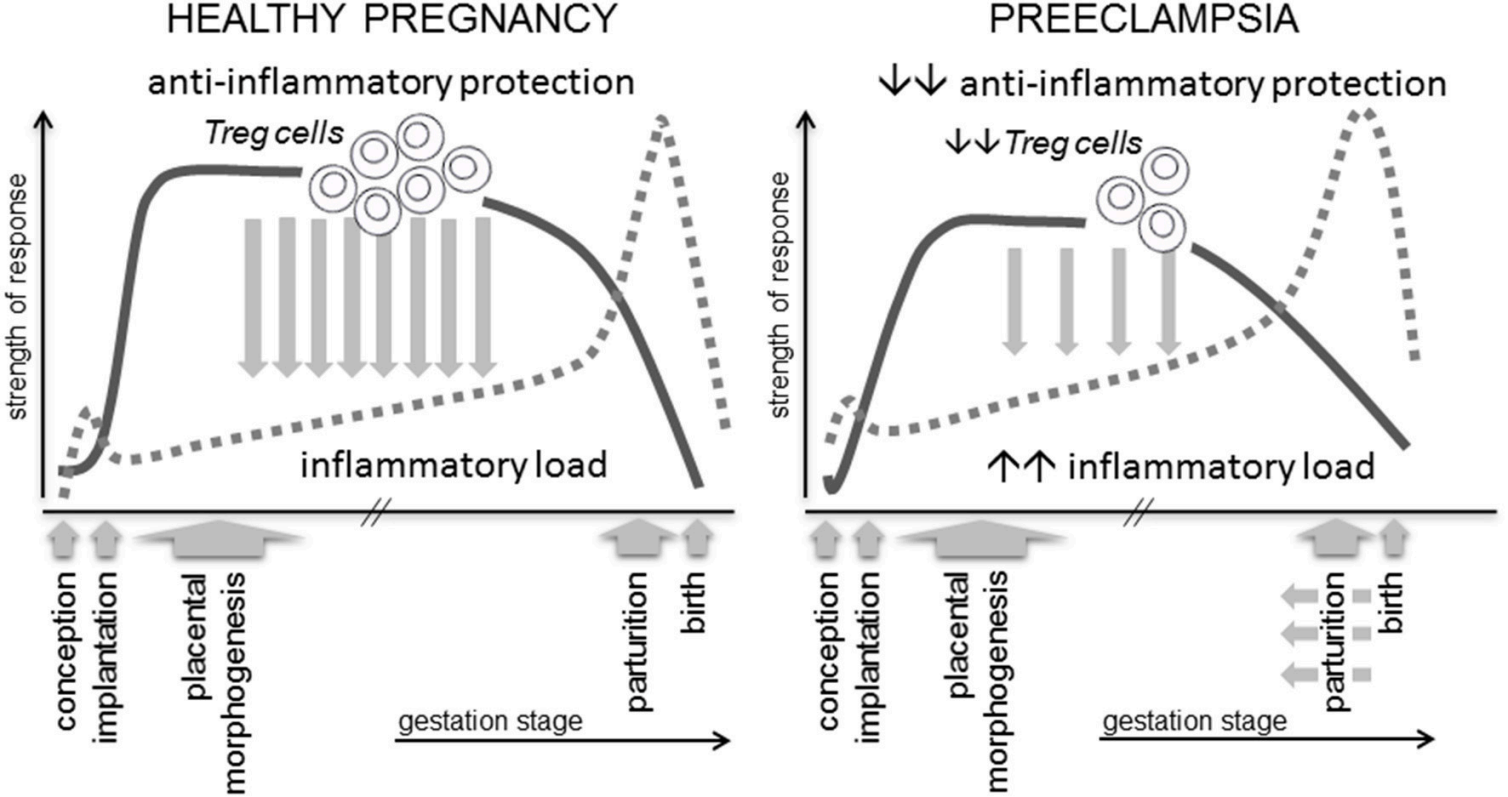
A robust immune response is essential for healthy pregnancy. Regulatory T cells (Treg cells) are a critical and rate-limiting element of the anti-inflammatory protection required to suppress inflammation and prevent adverse effects of anti-fetal effector immune responses. Treg cells arise as a consequence of an inflammation-like response during the peri-conception phase, and their abundance, suppressive function and stability are influenced by events prior to and during early pregnancy. Insufficient numbers, reduced suppressive function or instability are linked with preeclampsia and IUGR, potentially mediated by insufficient Treg cell capacity to support normal placental development and to suppress the elevated inflammatory load typical of this condition.

All women show evidence of altered immunity and elevated inflammatory activation in pregnancy. Immune adaptation for pregnancy commences during the pre-implantation phase when conception and implantation evoke a controlled inflammatory response in the female reproductive tract, which must be rapidly resolved by specific cytokines and pro-tolerogenic mechanisms into an anti-inflammatory milieu, in order for pregnancy to progress (23). In preeclampsia there are excessive pro-inflammatory mediators and inappropriate activation of effector immune cells, detectable in peripheral blood and gestational tissues from the first trimester (24, 25), implying incomplete or insufficient establishment of anti-inflammatory mechanisms (Figure 1).

In healthy pregnancy, inflammation associated with conception and implantation is rapidly resolved, and then remains suppressed by anti-inflammatory protective mechanisms, amongst which the specialized subset of CD4^+^ T lymphocytes called regulatory T cells (Treg cells), are pivotal (26–28). Through their critical roles in constraining inflammation, suppressing effector immunity, and modulating vascular function, Treg cells are emerging along with uNK cells and macrophages as key coordinators of implantation and early placental development (23, 29–31).

In preeclampsia, Tregs in the maternal peripheral blood and decidua are fewer in number (26, 28, 32) and their suppressive function is impaired (33, 34), while pro-inflammatory Th17 cells (27), CD8^+^ effector T cells and trophoblast apoptosis (28) are increased. The underlying reasons are unclear, but number and functional capacity of Treg cells are known to vary between individuals and are influenced by agents and exposures identified as pre-pregnancy antecedents of preeclampsia and other adverse pregnancy outcomes. Risk factors for impaired Treg activity include elevated inflammatory load associated with obesity and metabolic dysfunction (35), autoimmune conditions and systemic inflammatory exposures (36, 37), nutritional deficiencies (38, 39), and age (40). Their abundance and phenotype in the uterus are furthermore regulated by relevant clinical factors including prior pregnancy, disparity between male and female partner alloantigens, and seminal fluid contact (23). Other obstetric disorders including fetal growth restriction, gestational diabetes, and spontaneous preterm birth also have an inflammatory etiology, but amongst these conditions, the causal link between Treg cell dysregulation and preeclampsia is most clear.

In this review, we make the case that interventions to boost the number, functional competence and stability of Treg cells may offer realistic preventative and therapeutic strategies to protect against preeclampsia in at-risk women. Several pharmacological agents and cell therapy approaches to target Treg cells are in clinical trials or under development for auto-immune disorders and organ transplantation (37, 41, 42). We argue that as Treg therapies move closer to reality in other clinical settings, these interventions warrant evaluation for their potential utility in preeclampsia and related obstetric disorders with an immune etiology.

## Treg Cells—Essential for Maternal Adaptation to Pregnancy

Several mechanisms of active immune tolerance arise in early pregnancy to dampen inflammation and suppress allo-reactive immune responses that otherwise threaten conceptus survival. These include attenuated expression of polymorphic MHC molecules on placental tissues, trophoblast production of anti-inflammatory and pro-tolerogenic cytokines and hormones, and epigenetic modulation of decidual cell chemokine expression to prevent effector T cells (Teff) cells accumulating at the maternal-placental interface (43–45).

Amongst the various mechanisms of maternal tolerance, CD4^+^ Treg cells are essential for embryo implantation and early placental development (46, 47). Their capacity to constrain and resolve the inflammation elicited during embryo implantation, and suppress generation of immune effector cells in local lymph nodes, is pivotal to controlling inflammation and promoting immune tolerance over the course of gestation, until an inflammatory shift emerges again at parturition (Figure 1). This function is consistent with essential roles for Treg cells in immune homeostasis throughout the body, where they prevent autoimmunity to self-antigens, suppress Teff cells reacting to non-dangerous foreign antigens, regulate and limit excessive inflammation (48–50), and have important roles in tissue repair and homeostasis (51).

Different subsets of T cells with regulatory functions exist. CD4^+^ Treg cells, CD8^+^ Treg cells, gamma/delta T cells, Tr1 cells, and NKT cells can all exert suppressive functions and appear to operate collaboratively to control immune responses. CD4^+^ Treg cells are of particular interest because of their strong association with preeclampsia, and their potential for therapeutic manipulation (41). CD4^+^ Treg cells comprise about 1–3% of total T helper cells in humans and 3–10% in mice, and are defined by their expression of the master transcription factor Forkhead Box P3 (FOXP3). As well as FOXP3, CD4^+^ Treg cells constitutively express surface molecules including the IL2 receptor α-chain (CD25), the immune checkpoint receptor cytotoxic T-lymphocyte protein 4 (CTLA4), and glucocorticoid-induced tumor necrosis factor receptor (GITR), and in humans are CD127^−^ or CD127^low^ (49, 52).

There are two types of CD4^+^ Treg cells (referred to hereon as “Treg cells”). Thymus-derived Treg cells (tTreg cells) emerge from the thymus after self antigen-driven selection as functional suppressor T cells. Peripheral Treg cells (pTreg cells) differentiate from naïve CD4^+^ precursors after contact with antigens in peripheral lymph nodes or tissues (52). Differentiation of naïve CD4^+^ T cells into pTreg cells requires cognate antigen to be presented by antigen-presenting cells (APCs) such as pro-tolerogenic dendritic cells (tDCs) in the presence of IL2 and TGFB. The CD4 cells are thereby induced to express FOXP3 and become committed to suppressive function (53). These cells then promote a cycle of *de novo* Treg cell generation and drive the development of long-lasting immunologic memory, which is reinforced by persistent antigen exposure (54). Like pTreg, tTregs can also be induced to proliferate and acquire greater suppressive function by antigen contact in the periphery (51, 55, 56). In humans, tTregs and pTregs are not readily distinguishable but in mice, tTregs express neuropilin 1 (Nrp1) while pTregs are generally Nrp1 low or negative (52).

pTreg cells and tTreg cells exert anti-inflammatory and immune suppressive activity by secreting a range of soluble factors including IL10 and TGFB, as well as through cell contact-dependent mechanisms. Importantly, Treg cell suppressive function inhibits proliferation and cytokine release from pro-inflammatory CD4^+^ Teff cells, T helper 1 (Th1) and T helper 17 (Th17) cells, which typically produce pro-inflammatory IFNG and IL17, respectively. Activated Treg cells interact with DCs through CTLA4, to cause down-regulation of DC co-stimulatory molecules CD80 and CD86, which drive Teff cell activation (49).

## Altered Treg Cells Accompany and may Precede Preeclampsia Onset in Women

In women, T cells comprise 10–20% of decidual immune cells in the first trimester (57). Many decidual T cells are CD8^+^, including regulatory subsets (58, 59). Amongst the CD4^+^ T cells, around 10–30% express FOXP3, which is a substantial enrichment compared to peripheral blood (60–62). The Tregs comprise of both tTregs and pTregs and exhibit heterogeneous phenotypes that vary across the menstrual cycle and phase of pregnancy (32, 63, 64).

There is substantial evidence that many pregnant women with preeclampsia have fewer and less functionally competent Treg cells, accompanied by increased Teff cell activity, particularly Th1 and Th17 cells in decidual tissue and peripheral blood (26–28, 34, 65, 66). In a recent meta-analysis, a total of 17 independent primary studies were evaluated, and all but 2 showed consistent evidence of association between both severe, early-onset and late onset preeclampsia with fewer Treg cells in the third trimester (67). As well as reduced numbers, the suppressive function of Treg cells is often compromised in preeclampsia (33, 34, 68). The decrease in Treg cells may be proportional to the severity of disease (26), although relationship with time of disease onset and co-incidence of fetal growth restriction have not been consistently documented. There is evidence of an altered balance in Treg cell subsets in preeclampsia, with reports of fewer peripheral blood naïve HLADR^neg^ CD45RA^+^ Treg cells (68, 69) and fewer CD45RA^+^CD31^+^ recent thymic emigrant Tregs (64) in peripheral blood. Decidual Treg populations may be differentially affected, given decidual tDCs exhibit a reduced capacity to induce pTreg in preeclampsia (32).

Treg cell changes become evident in peripheral blood and gestational tissues shortly after conception and accumulate in decidua reaching their highest levels in early to mid-gestation, before decreasing as term approaches (28, 61, 70). A recent study utilizing chorionic villous sampling (CVS) at week 10–12 of gestation, showed that women who progress to preeclampsia demonstrate dysregulated expression of decidual and immune cell genes from this early time (71). In another study, elevated expression of IL6 which counteracts Treg stability and promotes Th17 generation (72), as well as reduced numbers of alternatively activated M2 macrophage and T cell markers, were detected in CVS tissues of women who later develop preeclampsia associated with fetal growth restriction (IUGR) (73). Although longitudinal studies to track Treg cells over the course of gestation are not yet reported in women with preeclampsia, there is good evidence that low abundance of circulating Treg cells in the first trimester is predictive of miscarriage before 12 weeks (74). Collectively, these observations underpin a working hypothesis that disturbed immune adaptation in early pregnancy precedes impaired placental development, setting the scene for later emergence of preeclampsia and related complications of pregnancy (8, 10, 29, 75).

This fits an emerging paradigm which positions early pregnancy as the origin of disorders of deep placentation that underpin early onset, severe preeclampsia, and also contribute to IUGR, preterm labor, premature rupture of membranes, and late spontaneous abortion (11, 76, 77). So-called shallow placentation arises from insufficient trophoblast invasion and failure to adequately remodel spiral arteries and to achieve high capacity maternal blood flow, which further compromises placental development and function, and leads to IUGR (1, 7, 8).

Treg cells are emerging as key regulators in the decidual leukocyte network which controls implantation and placental development. Through interactive cross-regulation, growth factor secretion and extracellular matrix remodeling, this network controls the decidual immune environment which facilitates trophoblast invasion and cytotrophoblast shell development, and enables remodeling of the decidual vasculature to support placental development (10, 78).

**Figure 2:**
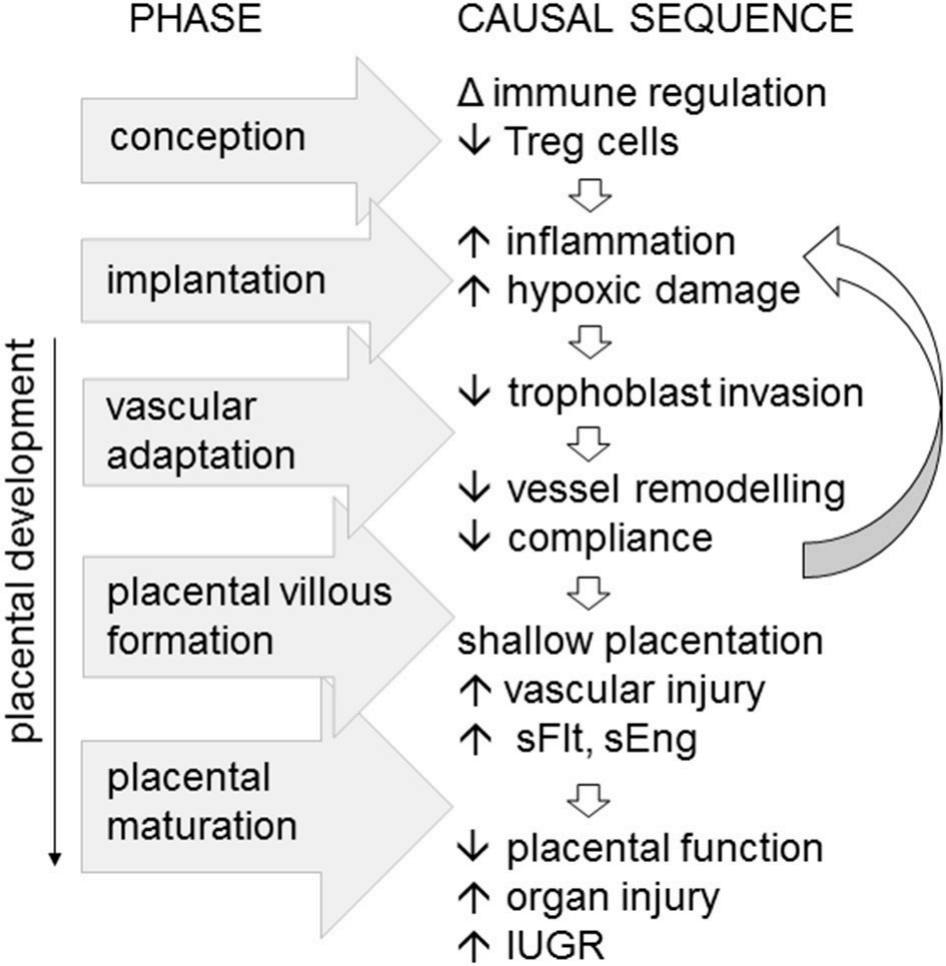
Immune imbalance associated with insufficient or incompetent Treg cells are implicated as an upstream cause of preeclampsia, particularly in severe, early onset disease. In a working model of the sequence of pathophysiological events, fewer Treg cells generated at conception impact the decidual environment at implantation, to limit trophoblast invasion, constrain maternal vascular adaptation, and vessel compliance. The resulting “shallow” placentation causes vascular inflammatory injury accompanied by elevated soluble Fms-like tyrosine kinase (sFlt) and soluble endoglin (eEng), reducing placental function, and causing maternal organ injury and *in utero* growth restriction (IUGR) of the fetus.

Inappropriate function or insufficient numbers of Treg cells in the decidua are linked with inadequate extravillous trophoblast invasion, and poor spiral artery remodeling, in turn destabilizing placental development and resulting in “shallow” placentation (12, 79). There is also a clear link between Treg deficiency and both recurrent implantation failure and recurrent pregnancy loss, where more severe forms of impaired uterine receptivity arrest trophoblast invasion and early placental development (80, 81). Thus, it is not difficult to envisage how insufficient Treg cells in the preconception and peri-conception phase could be a key upstream trigger for the sequence of events leading to impaired vessel remodeling and shallow placentation, which ultimately cause the overt symptoms of preeclampsia in later gestation (Figure 2).

## Treg cell Regulation of the Decidual Immune Environment

Mouse models have been instrumental for defining mechanisms through which Treg cells exert anti-inflammatory activity to influence the decidual environment and early placental development. Kinetic studies show that Treg cells accumulate in the uterine decidua from very early in pregnancy, and that these originate after naïve T cell activation and proliferation in local lymph nodes, causing numbers to expand through the first half of gestation (20, 46). After recruitment into the implantation site, Treg cells comprise around 30% of decidual T cells in the mouse (46).

Extensive experiments wherein Treg cells are selectively depleted, or overwhelmed by exacerbated Teff cell responses, show an essential role for Treg cells in preventing generation of destructive immunity to fetal alloantigens (82–85). Without sufficient Treg cells, an aggressive Th1 and Th17 mediated-response causes fetal loss in allogeneic but not syngeneic pregnancy (46).

Depending on the severity and timing of manipulation, Treg depletion can manifest as implantation failure, miscarriage or fetal growth restriction. Several studies show the pre- and peri-implantation phase is highly vulnerable. Administration of anti-CD25 Ab before or shortly after mating causes complete implantation failure (86–88). Depleting FOXP3^+^ cells from *FOXP3-Dtr* mice during early placentation increases later fetal resorption (85, 89), but depletion in mid-gestation only moderately reduces fetal viability (20), unless mice receive a second hit inflammatory challenge such that Treg depletion exacerbates the adverse impact (90–92). Mice deficient in T cells due to *Rag1*-null mutation are highly vulnerable to inflammation-induced fetal loss, but this is reversed by administering CD4^+^ T cells that differentiate to Tregs after transfer (90). Midgestation depletion of CD25^+^ cells using anti-CD25 mAb has a less severe impact than in early pregnancy, but this may be because Teff cells are also removed (86, 93). Additionally, other tolerogenic mechanisms including IL10 secretion by uNK cells (94) may compensate for Treg deficiency once placental development is complete.

Mouse models with a high rate of spontaneous fetal loss also demonstrate a critical role for Tregs in embryo implantation. CBA/J females mated with DBA/2J males have fewer decidual Tregs and elevated Th1 cells (87, 95). Adoptive transfer of Tregs from donor CBA/J females mated with Balb/c males boosts decidual Tregs and corrects fetal loss (87), but only if Treg transfer occurs before embryo implantation (87). These findings confirm that Tregs are most essential in the uterus during the peri-implantation period, consistent with a central role in orchestrating the transition to an anti-inflammatory mileu required for placental development (Figure 1).

Treg cells co-localize in clusters with uNK cells and other leukocytes in the human decidua basalis (78), where they exhibit activity expected to potently influence the local immune environment by enforcing an anti-inflammatory phenotype in other leukocyte lineages. In particular Tregs regulate uNK phenotype, through releasing TGFB and IL10 to control DC release of uNK viability factor IL15 (96), and suppress uNK cytolytic activity (91, 97). This may be particularly important in first pregnancy, given that uNK cells acquire memory and assume a more differentiated “trained” phenotype in subsequent pregnancies (21). Whether there is an interaction between antigen-experienced Tregs and trained uNK cells, remains to be investigated.

Treg cells also regulate M2 macrophages (98), mast cells (99), and tDCs, releasing heme oxygenase-1 which maintains immature DCs (100) and promotes indoleamine 2,3-dioxygenase (IDO) production to impair Th1 cell survival (101, 102). M2 macrophages and tDCs promote further Treg generation (98, 100) and produce an array of cytokines that reinforce a pro-tolerogenic decidual environment, including TGFB, CSF2 (GMCSF), IL4, IL10, CSF3 (GCSF), and prostaglandin E (103). Decidual Tregs also express other hallmark mediators of Teff suppression CD25, CTLA4, and PD-L1 (61, 91, 104–106).

Uterine NK cells and DCs are implicated as key regulators of decidual transformation (107–109) so through regulating uterine DC and uNK phenotype, Tregs would indirectly influence the extent and quality of the decidual response. Furthermore, trophoblasts engage with Tregs in a reciprocal interaction to modulate the secretory profile of both lineages (110). Together, these coordinated interactions allow Tregs to constrain inflammation and limit oxidative stress caused by trophoblast invasion during early placental development (13, 25, 111).

## Treg cells Influence Maternal Vascular Remodeling and Early Placental Development

**Figure 3:**
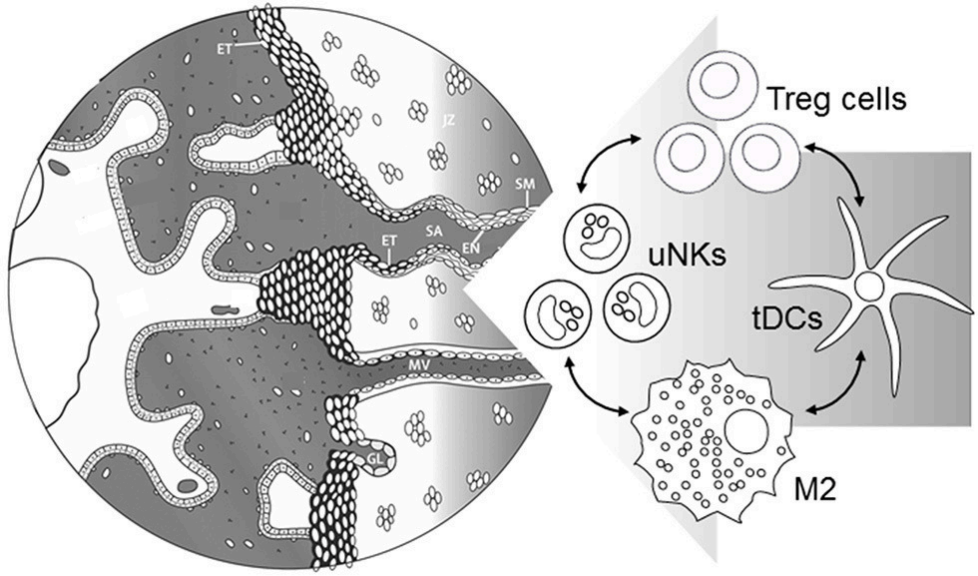
Events during early placental development require immune cells in the decidua to support maternal blood vessel (MV) adaptation and spiral artery (SA) transformation. Treg cells interact with uNK cells, M2 macrophages, and tDCs to suppress inflammation and provide secretory factors regulating extravillous trophoblast (ET) invasion, and functional changes in endothelial cells (EN) and surrounding smooth muscle (SM). Severe early-onset preeclampsia is accompanied by insufficient Treg cells, which in turn would be expected to contribute directly and indirectly through immune cell networks to impair vascular adaptations required for robust placental development. Left hand part of Figure is adapted from Steegers et al. (1).

Treg cells are emerging as critical participants in the process of maternal vascular remodeling, through their modulating effects on the decidual leukocyte network (Figure 3). There is extensive evidence to demonstrate key roles for uNK cells (30, 112), macrophages (31), and mast cells (99) in decidual vessel transformation, and in collaborating with invading trophoblasts to restructure the endothelial surface and smooth muscle wall (7, 13, 104). As detailed above, Treg cells exert potent anti-inflammatory actions on uNK cells (91, 97), M2 macrophages (98), mast cells (99), and tDCs (100), thereby influencing the vascular remodeling process.

This is unsurprising given growing evidence that Treg cells play important roles in modulating cardiovascular function, and vascular homeostasis throughout the body (113). In hypertensive mouse models, Treg cell infusion reduces blood pressure and vascular damage, and reverses hypertensive sequelae (114, 115). Treg cell-derived cytokines, particularly IL10 and TGFB, suppress inflammatory endothelial cell activation and inhibit development of atherosclerosis (113).

Rodent models of preeclampsia support a critical function for Treg cells in the pathophysiological events underlying abnormal placental development, through coordinated interactions with uNK cells, DCs, and macrophages (Figure 3). Experiments in mice deficient in T cells and/or NK cells show that T cells interact with uNK cells to influence the maternal hemodynamic response to pregnancy (116, 117). When cause-and-effect relationships are explored by antibody-mediated or genetic modulation of T cell subsets, Treg cells are implicated as having causal roles in the maternal and fetal symptoms of preeclampsia models.

Treg-deficient mice consistently show impaired uterine spiral arterial modification, reduced placental blood flow, and fetal growth restriction (85, 89, 118). Depletion of FOXP3^+^ Tregs in early pregnancy causes later dysfunction in uterine arteries accompanied by increased endothelin-1 production (89). Peripheral Treg cells are particularly implicated, as indicated by experiments in mice with a null mutation in the CNS1 gene which is a *FOXP3* enhancer element essential for pTreg cell but dispensable for tTreg generation. CNS1 is only present in eutherian mammals, suggesting its introduction into the FOXP3 locus to enable pTreg generation, in turn facilitated evolution of placentation (85). In CNS1-null mice, impaired remodeling of material spiral arteries underpins defective placental development (85). Compromised trophoblast invasion and failed transformation of spiral arteries is also seen in mice where neutrophil depletion causes insufficiency of pro-angiogenic, neutrophil-induced Treg cells (118).

In the reduced uterine perfusion pressure (RUPP) model of preeclampsia in rats, reduced uterine artery flow is induced by clip placement on the abdominal aorta and right and left uterine artery arcades at day 14 of gestation, resulting in placental ischemia and oxidative stress. The model replicates human preeclampsia symptoms with hypertension accompanied by increased circulating VEGF, sEng, Flt1, and placental growth factor (PlGF), plus elevated inflammatory cytokines and IUGR. A substantial (~50%) reduction in decidual and placental Treg cells, and elevation in total CD4^+^ T cells and Th17 cells, is a consequence of the RUPP intervention. Remarkably, the preeclampsia symptoms induced in this model are T cell dependent since the RUPP intervention does not cause hypertension and IUGR in T cell deficient athymic rats, and disease can be induced by passive transfer of Th17 effector CD4^+^ T cells (119). Treg cell deficiency is a key driver of hypertension and IUGR and these symptoms are mitigated when Treg cells from pregnant control donor rats are administered shortly after the RUPP procedure (120). Treatment strategies applied to boost endogenous Treg cells, including IL10 administration (121) or low dose CD28 superagonist (120), also reduce hypertension and IUGR.

Rodent models show that the protective effects of Treg cells are crucial from the early implantation phase, when vascular adaptation and early placental development begin. Several studies using different approaches to deplete Treg cells at various time points show the peri-implantation phase is most severely affected, with extensive Treg cell depletion at embryo implantation causing complete implantation failure (46, 86, 87). Experiments in the abortion-prone CBA/J x DBA/2 mouse model indicate transferred Treg cells can rescue the underlying placental defect, but only if Treg cells are transferred from healthy pregnant mice at or before the time of embryo implantation (87). Treg cell replacement influences other immune cells in the decidua, including mast cells, to repair placental and vascular defects and prevent sFlt elevation and fetal loss (99). Consistent with a critical role for peri-implantation Treg cells, a genetic model of preeclampsia involving overexpression of human angiotensinogen and renin in rats showed greater responsiveness to Treg cell therapy when it was applied in early gestation (122).

Even subtle disturbances to the T cell response in early pregnancy may impact later pregnancy progression. This may be the consequence of an altered environment during T cell activation, as we have recently demonstrated for CD8^+^ T cells in pregnancy (123). Other studies in mice show that reduced numbers or altered function of Treg cells at conception can disrupt fetal-placental development without immediate adverse effects, but with a legacy that becomes apparent in mid- or late gestation, particularly when a “second-hit” inflammatory challenge is applied (106, 124).

## The Treg Cell Response is Determined in the Pre- and Peri-conception Phase

The conditions under which the Treg cell populations of pregnancy originate are likely to be critical to the reduced quantity and impaired quality of the Treg response in preeclampsia. Uterine recruitment of Tregs in readiness for possible embryo implantation commences in the proliferative phase of each cycle, with an estrogen-driven increase peaking around ovulation (125). CD4^+^ FOXP3^+^ cells, thought to be pTregs based on expression of the Helios marker, are a major subset amongst the expanding Treg populations in blood and decidua in early human pregnancy (32). Helios^+^ Treg cells appear to be preferentially recruited into the decidua in the first trimester (63). Amongst peripheral blood tTregs the population of CD45RA^+^CD31^+^ cells, which have recently emigrated from the thymus, expand prominently in the first trimester and differentiate into CD45RA^−^CD31^−^ memory Tregs (64).

The majority of decidual T cells in women have a memory phenotype (CD45RA^−^ or CD45RO^+^) (59, 126) and show evidence of fetal antigen specificity (62), which indicates antigen exposure must occur to elicit the full Treg cell response. HLA-C is the only polymorphic HLA expressed by human placental trophoblasts, and fetal-maternal HLA-C mismatch is associated with a greater expansion in decidual Tregs (127). Many decidual Tregs show fetal HLA-C antigen specificity (62, 128), but whether other reproductive or tissue antigens are involved has not been investigated.

In preeclampsia, Treg cell deficiency is most pronounced in pTreg cells (32), as well as CD45RA^+^CD31^+^ recent thymic emigrant tTregs less able to acquire a memory phenotype (64). This implies there may be an underlying problem with antigen priming. Consistent with this, dysfunctional DCs with reduced HLA-G and ILT4 (32), and/or insufficent PD-L1 (129), have been reported in preeclampsia.

Contact with fetal alloantigens must occur under conditions that favor antigen presentation and stable Treg cell (not Teff cell) development. These conditions occur in two waves in the reproductive process. Paternally-derived transplantation antigens shared by the fetus are first and most frequently contacted during transmission of seminal fluid at coitus, at conception and in pre-conception cycles (22). Seminal fluid primes the activation of pTregs that are specific for paternal transplantation antigens which will later be expressed by fetal and placental cells. Additionally, once pregnancy is established and maternal blood comes into contact with the syncytiotrophoblast surface, placental exosomes are released into maternal blood, providing a second wave of alloantigen exposure (130, 131).

Again, mouse models have been informative in tracing the origins and regulation of Treg cells and point to specific events as critical for generation of the Treg cell pool in early pregnancy (132). The two stages of T cell activation can be tracked through the first half of gestation using T cell transgenic mice (93). The strength of seminal fluid as the initial priming event is first seen as a burst of T cell proliferation in the peri-conception phase, evident in cells recovered from the uterus-draining para-aortic lymph nodes (dLN) on day 3.5 post-coitus (pc), followed by a steady progressive increase during the post-implantation phase once placental morphogenesis is complete (93).

The first wave of proliferation of Treg cells can be detected within days of insemination in the lymph nodes draining the reproductive tract, in the peripheral blood, and spleen (46, 133). Seminal fluid contains paternal alloantigens and high levels of TGFB, and elicits an inflammation-like response in female reproductive tract tissues. DCs and macrophages recruited into female tissues take up seminal fluid alloantigens, traffic to the dLN and present antigen to naive T cells (93). Treg expansion is maximized in allogeneic compared with syngeneic matings, demonstrating a contribution of male alloantigens (134), but endogenous antigens might also contribute to the activation and expansion of Tregs in early pregnancy (135). Amongst the responding pTregs, paternal antigen-reactive pTreg cells are selectively enriched (133, 136). Data from mice with a mutation in the CNS1 gene show elevated fetal loss when pTreg alone are deficient, suggesting that pTreg cells have non-redundant functions important for viviparous pregnancy (85).

A population of tTreg cells of thymic origin also expand systemically prior to conception. These cells are recruited into the uterus after proliferation in the dLN during the estrous stage of the reproductive cycle in mice, in response to rising estrogen at ovulation (125, 137). After mating, factors in seminal fluid induce tTreg to proliferate and express elevated FOXP3 and CTLA4, both markers of suppressive competence, accompanied by demethylation of the Treg-specific demethylation region (TSDR) in the *FOXP3* locus (138). This expansion of tTreg cells occurs in parallel with the seminal fluid antigen-driven expansion of pTreg cells. This population may well have different functional qualities to pTreg, although these are still to be defined.

After recirculation via peripheral blood, Treg cells are recruited into the fetal-maternal interface in response to chemokines secreted by uterine epithelial cells including CCL19 (133), and may be stimulated to undergo further rounds of proliferation locally in the uterine tissue (46). The resulting expansion of the Treg cell pool induces a state of hypo-responsiveness to paternal alloantigens, concurrent with embryo implantation when the conceptus first contacts maternal tissues (136, 139). The kinetics of Treg cell induction in the peri-conception phase ensures sufficient abundance of Treg cells in the endometrium at embryo implantation, when their function is most critical (86, 87, 140). Continued release of paternally-inherited alloantigen from trophoblasts over the course of pregnancy sustains the T cell response until post-partum (20, 93). After birth, a population of paternal alloantigen-reactive Tregs are sustained, and in the event of a subsequent mating with a male expressing the same alloantigens, there is accelerated expansion of Treg cells driven by proliferation of fetal-specific Treg cells retained from the prior pregnancy (20).

Mouse studies imply that the immune response initiated at seminal fluid priming is a crucial initiating step and highly vulnerable phase for Treg cell tolerance to be established. In particular, responding pTreg cells require appropriate environmental signals including the cytokines IL2 and TGFB, to ensure naïve T cells differentiate into Treg cells and not Th1 or Th17 effector T cells (93, 133). Both the size of the Treg cell pool and the suppressive competence of pTreg cells will be determined by the strength of the antigenic challenge, and the nature of the cytokine context in which antigen contact occurs—parameters which are determined by seminal fluid composition as well as female tract factors. Since newly generated pTreg cells that have only recently commenced FOXP3 expression appear more vulnerable to phenotype switching and lineage instability (141), the extent to which pTreg cells primed at coitus will commit to a secure Treg fate will be substantially influenced by the conception environment. Relevant factors impacting this environment would include MHC disparity between male and female partners, the abundance and phenotype of DCs involved in antigen presentation, and bioavailability of local cytokines, hormones and other positive and negative regulators including microRNAs and the local microbiome (23).

Similar events occur in women, where the cervical immune response to seminal fluid mirrors the mouse response, causing elevated cytokine production, recruitment of leukocytes and T cell activation (22, 142), consistent with prior seminal fluid contact contributing to priming the paternal antigen-specific Treg cell response of pregnancy (62). It is yet to be proven that seminal fluid induces pTreg cells in women, and other factors must contribute to uterine Treg accumulation since IVF pregnancy can be established without seminal plasma contact. Expansion of uterine Tregs after conception may be further facilitated by human chorionic gonadotropin (hCG) secreted by invading placental trophoblasts (143). This builds on the hormone-driven expansion of Treg cells in the follicular phase of the menstrual cycle, correlating with progressively elevating serum E2 levels (125). However, the *in vivo* cervical response and an array of *in vitro* studies demonstrating that seminal fluid skews DC cells to an tDC phenotype and induces Treg cells *in vitro*, is consistent with a key role for seminal fluid in women (22, 144, 145). A priming effect of seminal fluid contact in women also explains the benefit of cumulative seminal fluid contact with the conceiving partner in protecting from preeclampsia (17, 146).

## Is Insufficient Priming a Cause of Treg Cell Deficiency in Preeclampsia?

An important question is why some women have fewer Treg cells and/or impaired Treg function at the outset of pregnancy. The nature and significance of factors that cause variation in the uterine Treg cell response are unclear and require investigation. As detailed above, antigen priming in the appropriate environmental context is a critical factor in the strength and quality of any peripheral tissue Treg cell response. In the reproductive tissues, the strength and quality of antigen and immune-regulatory signals in the female reproductive mucosa during priming would be paramount, as well as the number and timing of prior exposures to the conceiving partner's seminal fluid and any previous pregnancies with that partner (25).

This raises the possibility that some women develop preeclampsia after conceiving without adequate prior priming to male partner alloantigens. It seems likely that pTregs reacting with paternal alloantigens would be more vulnerable than tTregs to variations in population size, antigen experience and memory, functional competence, and stability. Because newly generated pTregs are particularly susceptible to phenotype-switching and lineage instability (141), the priming environment would be a key determinant of a secure fate amongst pTreg with male partner alloantigen specificity. Recent evidence in mice that seminal fluid contact regulates tTreg cells, inducing proliferation and reinforcing a suppressive phenotype through epigenetic modulation, suggests tTreg as well as pTreg are impacted (138).

Priming may be dysregulated due to seminal fluid composition or female responsiveness to seminal fluid signals (22, 25). It has been shown that recurrent miscarriage patients produce more CD4^+^IL17^+^ and CD4^+^IFNG^+^ cells and fewer CD4^+^CD25^+^FOXP3^+^ Tregs, compared to fertile controls, when CD4^+^ T cells are cultured with DCs and partner's seminal fluid antigens (147). The balance of immune-regulatory agents in seminal fluid, particularly pro-tolerogenic TGFB, varies between men, and within men over time (148). The anti-tolerogenic cytokine IFNG, which drives generation of Th1 immunity, fluctuates substantially and can become elevated in seminal fluid in the event of infection or other inflammatory conditions (149). IFNG interferes with synthesis of CSF2 required to drive the T cell proliferative response at conception (150, 151), skews Th0 differentiation toward Th17 cells (48, 152), and increases Treg susceptibility to transdifferentiate into Th17 cells (153).

## Clinical and Lifestyle Factors Impacting the Treg Cell Response

A suboptimal Treg cell adaptation for pregnancy could also occur in women due to intrinsic Treg deficiency. The specific factors determining between individual variation in Treg numbers and functional capacity are yet to be fully defined. An interaction between genetic, epigenetic, and environmental factors seems likely, based on data from animal models and limited studies in population cohorts (154). The thymic output of tTreg, and peripheral tissue induction of pTreg cells, are independently regulated and can be affected by a range of metabolic and nutritional parameters, inflammatory exposures, autoimmune conditions, and age (35–40). Common health conditions that affect the immune system including intestinal microbial dysbiosis and dietary deficiencies, particularly vitamin A and vitamin D, have been associated with poor adaptive immunity and may be a common cause of compromised Treg activity (38). Exposure to sunlight (55) and exercise (56) are also recently identified to support Treg homeostasis, but how these are linked to variation in human Treg parameters are yet to be defined.

In hyper-inflammatory conditions caused by autoimmune, infectious or metabolic disorders some pTreg cells exhibit phenotypic plasticity and instability, with increased disposition to shift phenotype, or lose *FOXP3* expression and become reprogrammed into a Teff fate (154). Studies in mice and humans demonstrate that FOXP3^+^ T cells can be induced by inflammatory stimuli to express IL17 and IFNG characteristic of Teff cells (155, 156), and may then transdifferentiate into effector Th17-like cells, known as “exTregs,” which can amplify inflammatory pathology (157).

Epigenetic regulation of *FOXP3* through demethylation of the TSDR region is a key factor in the resilience of Tregs to inflammatory stress, that controls whether T cells can express sufficient *FOXP3* to overrule Teff functions and maintain a Treg suppressive phenotype (158). Increased TSDR methylation and associated underexpression of *FOXP3* is a feature of some autoimmune conditions (159).

There is little information on whether Treg cells exhibit signaling defects, lineage instability or methylation changes in preeclampsia. Given the evidence of elevated Th1 and Th17 cells counteracting the decrease in Treg number and function in preeclampsia, defects in both Treg cell induction and/or stability seem plausible, and would explain the concurrent reduction in Treg cell suppressive function (34, 68). Although large populations have not been examined, exploratory studies in preeclampsia suggest an elevated incidence of gene variants within the promoter region of *FOXP3* that may affect expression levels and hence Treg stability (160, 161). Elevated IL6 trans-signaling, which is known to promote Treg instability and transdifferentiation, has been described in women with recurrent miscarriage (162). IL6 is associated with reduced TGFB output and IL2-mediated STAT5 signaling (163), and is a possible candidate contributing to impaired Treg capacity in preeclampsia, given that elevated expression of IL6 is seen in gestational tissues of women who later develop the condition (73).

## Potential Interventions to Target Treg Cells in Pregnancy

Recognition that excessive inflammation secondary to insufficient anti-inflammatory protection is a key driver of preeclampsia gives rise to the prospect of targeting the immune response to prevent or suppress progression of the disease. In particular, Treg cells provide an attractive target, because of (1) the clear link between compromised Treg cells and preeclampsia; (2) a logical mechanistic pathway placing insufficient Treg cells as an upstream event in the placental and systemic pathophysiological sequalae; (3) compelling evidence from preclinical rodent models showing that insufficient Treg cells can elicit preeclampsia-like symptoms, while boosting Treg cells mitigates symptoms, and (4) encouraging progress in development of Treg cell therapies for other autoimmune and inflammatory conditions.

Interventions to boost Treg cell populations and their suppressive competence are under development and show promise in autoimmunity and tissue transplantation (41, 42), and more recently have been considered for cardiovascular disease (113). Treg cell therapies relevant to preeclampsia could take one of three alternative approaches: (1) lifestyle and health advice during preconception planning to assist immune adaptation to pregnancy; (2) neutraceutical, pharmacological, or other strategies to increase Treg cell numbers and/or function in an antigen non-specific, systemic manner, or (3) cell therapy treatments that involve *ex vivo* generation and/or expanding Treg cells in a highly-individualized process. These clearly represent different degrees of technical challenge, invasiveness, cost and risk. While lifestyle adjustments or dietary supplements are generally safe and tractable, cell therapies are labor-intensive, expensive, and higher risk.

The evidence base for approaches to target Tregs in other clinical settings is building (41, 42), but to date little consideration has been given to applications in reproductive conditions. To advance new treatments targeting Treg cells for preeclampsia prevention and mitigation, research on several fronts is required. Most immediate goals should be to develop appropriate diagnostics, and to investigate and validate pre-pregnancy planning interventions to boost Treg cells. There should also be careful consideration of the rationale for initiating clinical studies, using robust clinical trial methodology, to evaluate pharmacological and/or cell therapy treatments for application when Treg deficiencies are not responsive to lower intervention approaches.

### Diagnosis of Treg Cell Deficiency

To progress understanding of Treg cell insufficiency in preeclampsia, and to develop therapeutic options targeting these, it is essential that effective diagnostic tools are developed and validated. These should detect common and informative defects in Treg cell parameters that define competency for healthy pregnancy, and ideally be applied to peripheral blood if preference to endometrial biopsies. Treg tests should be appropriate for routine use during pregnancy planning or early after conception, to provide a therapeutic window for early treatment interventions to prevent progression to miscarriage or later obstetric conditions. To date little work has been done to investigate Treg cell deficiency before conception, or in early pregnancy, in the blood or endometrium of women who go on to develop preeclampsia. Ongoing studies to address the pre-pregnancy origins of preeclampsia may begin to address this (9).

A recent meta-analysis of Treg cell parameters quantified in preeclampsia highlights the considerable variability in markers that different groups have measured to date (67). A useful step will be to develop a consensus definition of minimum essential Treg markers to facilitate harmonization across future studies, and to determine the best stage in preconception cycles or early pregnancy for analysis (164). Given the significance of tTreg cells vs. pTreg cells, and of naïve vs. memory cells in preeclampsia (68, 69), extensive marker panels to discriminate these subsets in flow cytometry-based tests will be most informative. Along with standard markers CD4, CD25, CD127, and FOXP3, markers that reflect memory, suppressive capacity, and activation status amongst Treg cells should be measured. These may include GITR which is emerging as a superior marker of active, functional Tregs (165), plus CTLA4, CD45RO, HLADR, and potentially intracellular cytokines or transcription factors which appear particularly informative in the preeclampsia setting (27, 34).

Ideally, Treg cell assays should also inform on suppressive competence in a paternal antigen-specific manner. Assessment of suppressive function by *in vitro*-based assays and/or analysis of *FOXP3* methylation status has been the gold standard for assessment of suppressive potential, but new markers such GITR, CD154, and PI16 may supersede these tests and be more amenable to a clinical diagnostic setting (165–167). With increasing availability of tetramer-based diagnostic tools for identifying TCR specificity, identification of partner alloantigen-reactive Treg cells may in time become feasible.

### Optimal Timing for Interventions

A major challenge for developing strategies to target Treg cells in pregnancy is timing—how and when would Treg cell deficiency be diagnosed, and how would this relate to the window of opportunity for intervention? With evidence that Treg cells are most critical during the implantation and early placentation phase of pregnancy, interventions in cycles prior to conception, or as soon as possible after conception would likely be most advantageous. The timing would need to align with hormone-driven regulation across the menstrual cycle, and might leverage events controlling estrogen and progesterone-regulated expansion of the Treg pool (125). Interventions would need to be coupled with Treg screening of high risk women during pre-pregnancy planning or early after conception to allow the best chance to identify patient subgroups that could be amenable to therapy.

### Treg Cells and Preconception Care

A tractable approach worthy of further investigation is advice on immune system health and boosting immune priming during preconception planning. In nulliparous women, the available evidence suggests on average, 3–6 months of sexual cohabitation without using barrier contraceptives is required for sufficient seminal fluid priming to minimize the chance of preeclampsia (15). Consistent with this, in a recent study of 340 women, women in the highest 10th percentile of exposure to partner's seminal fluid had a 70% reduced odds of preeclampsia relative to women in the lowest 25th percentile (168). Thus, advising nulliparous women to avoid use of barrier contraceptive methods and to consider increasing vaginal coitus prior to conceiving may reduce preeclampsia risk. A key question is the impact of different non-barrier approaches to facilitating immune priming, such as oral contraceptive pill or intrauterine device, which both deliver immune-modulating hormones. Further studies are required to evaluate the impact on preeclampsia rates of preconception advice on seminal fluid contact and contraceptive choice, and to investigate whether a partner-specific Treg cell response is involved.

Detecting and correcting any immune imbalance due to clinical, nutritional and/or, lifestyle factors is also likely to be effective for pregnancy planning and reducing susceptibility to preeclampsia. Elevated inflammatory load due to chronic infection, smoking, diabetic and pre-diabetic conditions, obesity and/or microbiome dysbiosis in women would be expected to adversely affect intrinsic Treg cell parameters and responsiveness to priming (35, 169), while in men these conditions may increase seminal fluid IFNG and reduce capacity to elicit a healthy female response (149). A range of autoimmune conditions known to impact reproductive function likely have a shared underlying etiology and correcting the immune disorder with validated approaches would reasonably yield dividends for pregnancy health (170). Microbiome disorders, and vitamin and micronutrient deficiencies also affect Treg cells, and treating these might have utility in boosting Treg cell activity in the reproductive setting, as has been shown for some other immune disorders (38). It will be important for future studies to trial the efficacy of alternative approaches to pre-pregnancy care, in order to determine the most effective interventions.

### Pharmaceutical Interventions to Expand Treg Cells for Pregnancy

High-risk women with a previous pre-eclamptic pregnancy are an obvious target for preconception care to boost immune tolerance. However, duration of sexual cohabitation is unlikely to be limiting in this patient group, and couple-intrinsic issues such as insufficient HLA disparity between partners, or HLA incompatibility resulting in low immunogenicity of male alloantigens, could theoretically interfere with priming and expansion of the Treg cell pool. In selected women with a demonstrated intrinsic Treg deficiency, approaches that target Treg cells might warrant consideration.

Agents of potential utility to induce Treg cell-mediated tolerance in women include cytokines and other biological agents. Two cytokines that been used clinically to attempt to enhance embryo implantation and placentation, CSF3 (171) and CSF2 (172), act on myeloid immune cells and promote recruitment and function of tDCs in the reproductive tract mucosa. Mouse studies are consistent with their fertility-promoting effects being mediated via tDC-mediated induction of Treg cells (151, 173), but their effect on T cells has not been studied in women. IL10 and several microRNAs that act to expand the Treg cell pool and increase functional competence, and are known to be induced naturally in the female tract response to seminal fluid, are also worthy of investigation (106, 174).

Several existing drugs deployed in pregnancy may act at least partly through Treg cells. Studies in mice suggest that progesterone mediates suppression of the Teff cell response, affecting CD4^+^ T cell and Treg cell phenotype (175, 176). Progesterone effectively suppresses the generation of Th1 cells and Th17 cells and induces Treg cell differentiation (177–179). Treg cells induced by progesterone have increased capacity to suppress the activation and expansion of Teff cells (177, 178). This fits with evidence of progesterone-regulated increases in uterine Treg cell populations in mice and in women (125, 137). Physiological levels of progesterone increase the functional population of CD4^+^FOXP3^+^ cells in pseudopregnant mice and increase the splenic CD4^+^FOXP3^+^ cell proportions in mid gestation (180). Progesterone also acts to selectively repress *IFNG* gene expression in CD4^+^ T cells (181), allowing enhanced induction of Treg cells and suppression of Th1 and Th17 differentiation (84, 182). A Cochrane meta-analysis demonstrated a benefit of progesterone for reducing recurrent miscarriage in women (183), but whether this impacts Treg cells is unknown. Furthermore, the outcomes in this setting are confounded by the large proportion of losses related to embryo chromosomal abnormalities, rather than immune dysregulation in the endometrium (184). There is no proven clinical benefit of progesterone in preeclampsia, and a Cochrane review did not find sufficient evidence to support its clinical use to prevent preeclampsia when administration was commenced between 16 and 28 weeks gestation in 4 clinical trials (185). Administration in early pregnancy would likely be required to improve Treg cells at the relevant developmental phase, but the effect of early administration of progesterone on susceptibility to preeclampsia has not been assessed.

Although immune suppressive glucocorticoid drugs conventionally used in autoimmune conditions are sometimes administered in assisted reproduction settings, these suppress both Treg cells and Teff cells, and carry risks when used in pregnancy (186). Intravenous immunoglobulins (IVIg) and Intralipid have also been empirically used in artificial reproductive technology settings to enhance implantation and in recurrent miscarriage clinics to reduce miscarriage rates (187). IVIg did not demonstrate an improvement in livebirth outcomes in 8 small studies in 303 women who suffered recurrent miscarriage (188). Although there is some evidence to suggest that intralipid infusions are associated with immune suppression and alter NK cell activity (189), their effects on Treg cell parameters has not been measured and their clinical benefit for implantation disorders or miscarriage is unproven in clinical trials. The impact of administering intralipid and IVIg infusion in early pregnancy on the development of early or late onset preeclampsia has not been assessed.

There are several drugs under development for autoimmune diseases, including immune checkpoint regulators and other immune-active biologics, that may afford greater selectivity for Treg cells than the immune modulating treatments described above (42). These approaches may be worthy of cautious evaluation in reproductive conditions. Drugs targeting immune checkpoint regulators CTLA4 and PD-1 offer enormous potential, and studies in preclinical models offer encouragement. A recent study in rats where preeclampsia-like symptoms are induced by L-NAME administration showed treatment with PD-L1-Fc protein was effective in reversing Treg/Th17 imbalance and mitigating placental damage (129). Substantial promise for a CD28 superagonist treatment was demonstrated in a rat model of preeclampsia induced by overexpression of human angiotensinogen. Administration of CD28 superagonist was highly effective in increasing Treg cells and alleviating maternal hypertension, proteinuria and IUGR, particularly when treatment was applied from the pre-implantation phase (122). Low dose IL2 has been used to expand Tregs in several conditions, including in abortion-prone mice where protection against fetal loss was achieved (135).

Other relevant agents include humanized antibodies against T cell markers such as anti-CD3, anti-CD52, and anti-CD45 RO/RA which reestablish immune tolerance by selectively depleting Teff cells and retaining Treg cells (37). Other approaches utilize cytokine specific monoclonal antibodies to promote Treg cells—these include anti-TNFA which is approved for use in rheumatoid arthritis and Crohn's Disease, or protolerogenic cytokines such as TGFB and IL10 (37).

Epigenetic regulation of *FOXP3* to impart elevated suppressive function and stability in Tregs is another candidate approach. Inhibitors of DNA methyltransferases such as 5-aza-2′-deoxycytidine (Aza), or factors involved in DNA methylation such as Ten-eleven translocation (TET) protein, have been utilized *in vitro* to drive hypomethylation of the *FOXP3* locus, resulting in strong, stable *FOXP3* expression in Treg cells (190–192). In mouse models, administration of DNMT inhibitors enhances Treg number, *FOXP3* expression and suppressive capacity which assists in reducing inflammation associated with LPS-induced lung injury (193), and prolongs cardiac allograft survival (194). Histone deacetylase (HDACs) inhibitors have also been shown to boost Treg cells and improve suppressive function, resulting in decreased inflammatory bowel disease and increase tissue graft survival in mice (195). These agents carry risks as well as potential, so any application in a reproductive setting would need to be carefully evaluated, initially in preclinical studies.

### Cell Therapy Interventions to Boost Treg Cells for Pregnancy

Cell therapy provides a challenging but highly personalized and thus potentially more effective approach to tackling Treg-mediated conditions (37). Cell therapy involves either (i) isolating *in vivo* differentiated Treg cells and expanding them *ex vivo* or (ii) generating and expanding pTreg cells *in vitro*, before subsequent reinfusion. These approaches are in development for transplantation and severe autoimmune disease, but would currently be difficult to justify for a non-life-threatening pregnancy condition. However, given that in preeclampsia the Teff response is not overwhelming, once Treg cell therapy becomes a reality it may prove to be more amenable than other conditions where an extreme immune deviation is beyond rescue (37, 41).

A substantial benefit of cell therapy is that antigen-specific Treg cells can be manipulated without systemic effects on the immune response, with lower risk of off target effects in the mother and fetus than with pharmacological approaches. Studies in type 1 diabetes and other diseases show that T cell receptor (TCR) reactivity with relevant antigens in the target tissue improve Treg cell recruitment and capacity to persist and execute effective suppression, with a low chance of non-specific immune suppression (41, 196). This is a challenge for many disease conditions that might be considered for Treg cell therapy, when Treg cells reactive to tissue-specific antigens are rare. However, the relevant antigens in pregnancy are paternal alloantigens where the starting frequency is much higher. A large proportion of naturally-occurring naïve CD4^+^ T cells, pTreg cells and tTreg cells react with allo-antigens and could readily be expanded amongst polyclonal populations. Furthermore, because of their capacity to suppress immune responses in an antigen non-specific manner (bystander suppression) and their capacity to skew T cell responses to other tissue antigens toward tolerance (infectious tolerance), it is possible to regulate the immune response in a whole organ using Treg cells reactive with a single antigen (37, 41). In pregnancy, this means that Treg cells reactive with just one or a subset of paternally-inherited fetal alloantigens, or perhaps even a male minor histocompatibility antigen such as H-Y, could reasonably be effective in suppressing immune responses to a wide range of placental and fetal antigens.

Enormous potential is offered by new gene modification developments in generating alloantigen-specific Treg cells using chimeric antigen receptor technology. This approach overcomes the challenge of the low frequency of antigen-reactive T cells occurring naturally in peripheral blood, by genetically manipulating Treg cells with self-specificity to express either a TCR complex, or a chimeric antigen receptor (CAR) reactive to specific antigens. Use of CAR technology can reliably generate potent, functionally competent, and stable alloantigen-specific human Treg cells that have utility in a wide range of human autoimmune diseases (197). Ongoing clinical trials are showing exciting results in Crohn's disease and are likely to soon be applied in the tissue transplant setting (198). There is a prospect that in time, obstetric disorders may be amongst the range of diseases to benefit from CAR T cell therapy—but again, this must wait until relevant reproductive antigens are identified, and can be targeted in a patient-specific manner.

## Conclusions

Immune imbalance or “maladaptation” has been implicated as central and causal in disease development in preeclampsia, and Treg cells are identified as a pivotal immune cell lineage. Their unique combination of anti-inflammatory, and immune modulatory properties affords Treg cells a potent capacity to support maternal vascular adaptation and placental development, suppress inflammation and sustain maternal tolerance of the fetus. The effects of Treg cells appear most critical at the time of pregnancy establishment and during early placental morphogenesis. Insufficient or dysfunctional Tregs provides a mechanism through which environmental, metabolic, and genetic factors can converge to increase disease risk (154), likely interacting with clinical factors such as prior pregnancy and immune compatibility between partners, which are known to be important pre-pregnancy antecedents of preeclampsia (10).

Given the rapid advances in Treg cell immunology including informative diagnostics based on flow cytometry of peripheral blood, and development of a range of low and high intervention treatments, the prospect of targeting Treg cells in at-risk women to treat early placental disturbances and effectively mitigate preeclampsia onset, warrants evaluation. It will be important to focus on developing diagnostics and interventions for application before or during early pregnancy, to divert the course of disease development before placental or fetal injury occurs. Proof-of-concept experiments in rodent models of preeclampsia already demonstrate the utility of biological agents PF-L1 Fc (129), CD28 superligand (122), and low dose IL2 (135) to boost Treg cell numbers and stability.

Experimental evaluation of any strategy to increase Tregs in a human reproductive setting must take a highly cautious approach and be founded in robust clinical trial design principles. It is critical that safety for mothers and infants is paramount, and the different risk-benefit ratio of reproductive and obstetric conditions, compared to life-threatening immune diseases, is recognized. Possible adverse consequences of artificially boosting maternal Treg cells, including reduced pathogen defense (199) or even reduced immune surveillance against malignancy (200) would need to be considered. Notwithstanding the substantial work to be done to evaluate alternative approaches and identify responsive patient groups, there is an imperative to invest in developing immune therapy options with the goal to reduce the morbidity and mortality associated with preeclampsia.

## Author Contributions

SR, EG, AC, and LM assembled and interpreted the relevant literature and prepared manuscript drafts and Figures. JP, MH, SB, and GD provided expert knowledge on content and revised manuscript drafts.

### Conflict of Interest Statement

SR receives income from Origio A/S. The remaining authors declare that the research was conducted in the absence of any commercial or financial relationships that could be construed as a potential conflict of interest.
